# Supplementary material for: Postmortem tissue biomarkers of menopausal transition
Source: Mol Psychiatry. 2025 Aug 27;31(2):819–34. doi: 10.1038/s41380-025-03177-9 (PMC12515968; doi:10.1038/s41380-025-03177-9)
Supplement: Supplementary file 1 — Supplementary Information [file 41380_2025_3177_MOESM1_ESM.pdf]

Supplementary Information

Postmortem tissue biomarkers of menopausal transition

Maria Tickerhoof[1], Heining Cham[2], Laila Ouldibbat[1], Anaya Ger[1], Sonola Burrja[1], Pavan Auluck[3], Peter J. Schmidt[4], Stefano Marenco[3], Marija Kundakovic[1]*

*Correspondence to: mkundakovic@fordham.edu

This file includes:

Supplementary Figures

Supplementary Figure 1: Correlation of blood vs. hypothalamic steroid measurements.

Supplementary Figure 2. Hypothalamic CYP19A1 gene expression and its correlation with blood and hypothalamic estradiol levels.

Supplementary Figure 3: Correlation of fourteen biological measurements with significant differences between premenopausal and postmenopausal samples.

Supplementary Figure 4: Principal component analysis for fourteen significant menopausal biomarkers.

Supplementary Figure 5: Correlation of eight biological measurements included in principal component analysis.

Supplementary Figure 6: Characterization of samples based on a composite measure including hypothalamic *CYP19A1* calculated by principal component analysis.

Supplementary Figure 7: Correlations of biomarkers included in tissue-specific component score calculations.

Supplementary Figure 8: Principal component analysis for tissue-specific component scores.

Supplementary Figure 9: Characterization of steroids-only blood component score.

Supplementary Figure 10: Supplemental PCA analyses with 7-marker model.

Supplementary Tables

Supplementary Table 1: Component score and classification versus manual characterization of menopausal status * Blank cell: not measured or missing; ND: Not detected; BQL: Below limit of quantitation. (uploaded as a separate Excel file)

Supplementary Table 2. Group values of all 40 measures analyzed by robust ANOVA.

Supplementary Table 3: Individual scores and classifications of all four tissue-specific component scores, the 8-marker component score, hypothalamic *CYP19A1* gene expression, and the 7-marker composite measure. (uploaded as a separate Excel file)

Supplementary Table 4: Blood vs. serum measures of AMH and steroids. (uploaded as a separate Excel file)

Supplementary Table 5: Primer sets used for qRT-PCR in the pituitary and hypothalamus.

Supplementary Figure 1

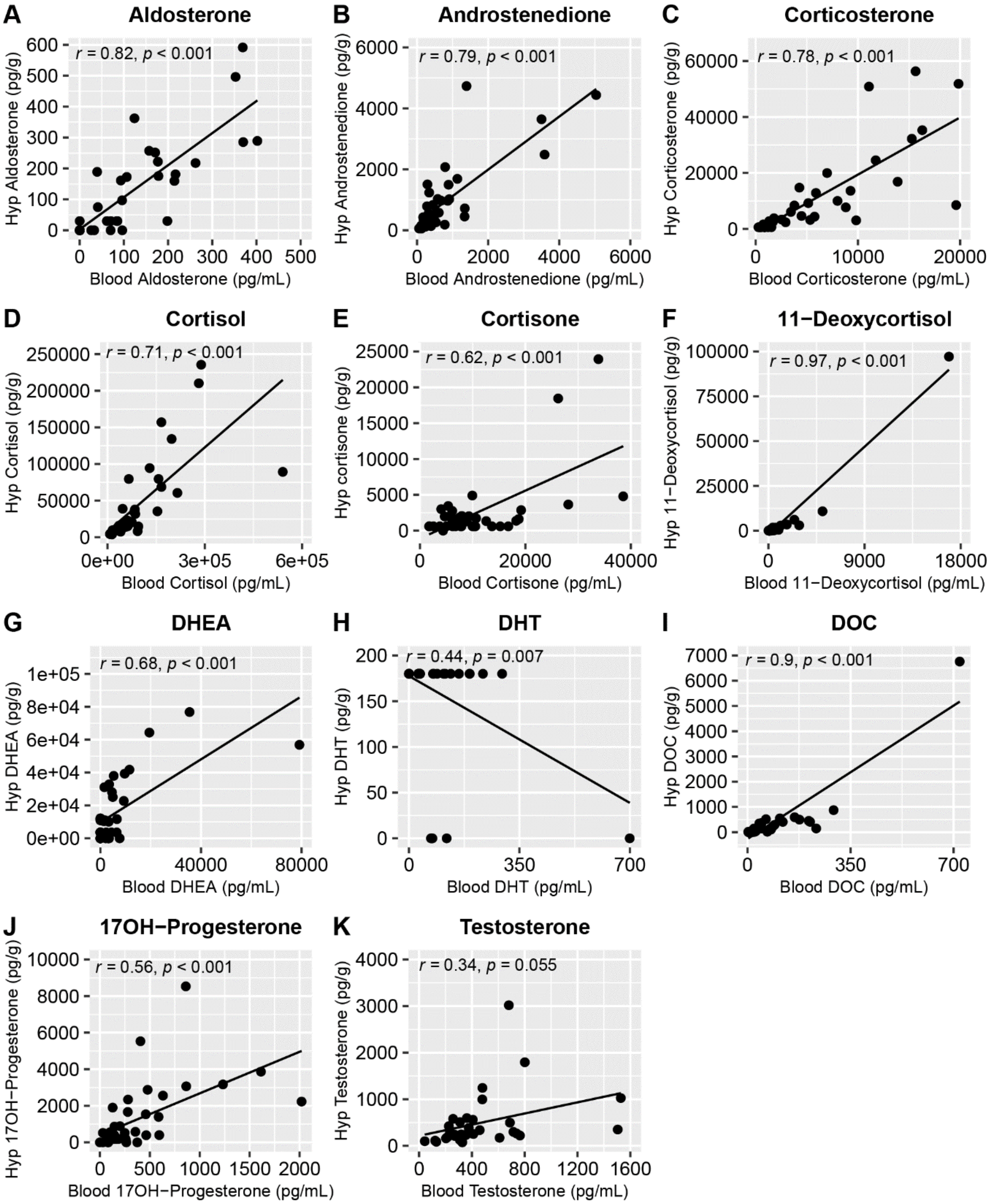

Correlation of blood vs. hypothalamic steroid measurements. All steroids, with the exception of DHT and testosterone, had significant moderate to very strong correlations between measurements from whole blood and measurements from hypothalamus tissue extract. Note that all hypothalamic DHT measurements were either undetectable (values replaced with 0 pg/g) or below the limit of quantitation (values replaced with ½ of the limit of quantitation). Glossary: DHEA – Dehydroepiandrosterone; DHT – Dihydrotestosterone; DOC – deoxycorticosterone.

Supplementary Figure 2

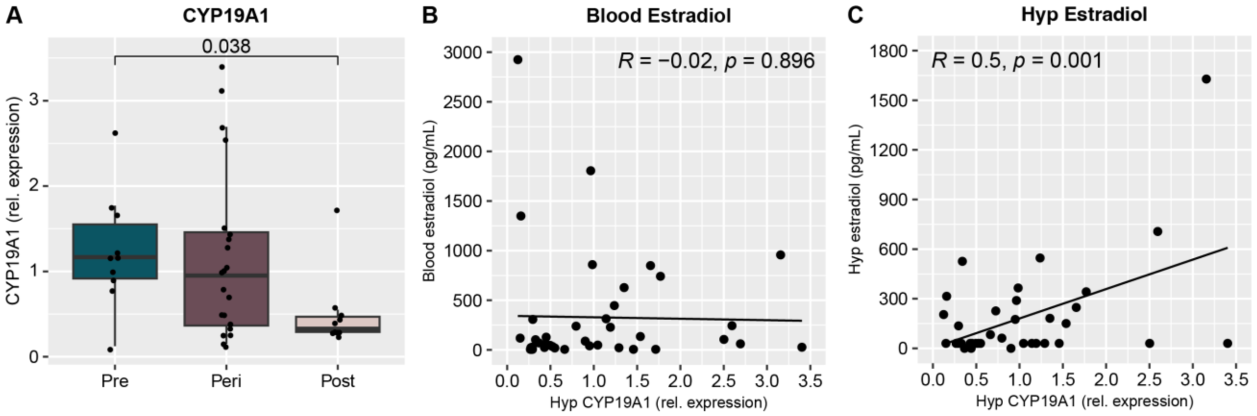

Hypothalamic CYP19A1 gene expression and its correlation with blood and hypothalamic estradiol levels. A. Relative gene expression of *CYP19A1* was analyzed in the hypothalamus of pre-, peri-, and post-menopausal groups. Hypothalamic *CYP19A1* gene expression was lower in the post-menopausal group compared to the pre-menopausal group. p value above the box plot indicates significance of Dunn's test posthoc comparison following non-parametric Kruskal-Wallis test. In addition, hypothalamic *CYP19A1*: **B.** does not correlate with blood estradiol levels, but **C.** correlates significantly with hypothalamic estradiol levels. Box plots (box, 1st-3rd quartile; horizontal line, median; whiskers, 1.5xIQR). Hyp, hypothalamic.

Supplementary Figure 3

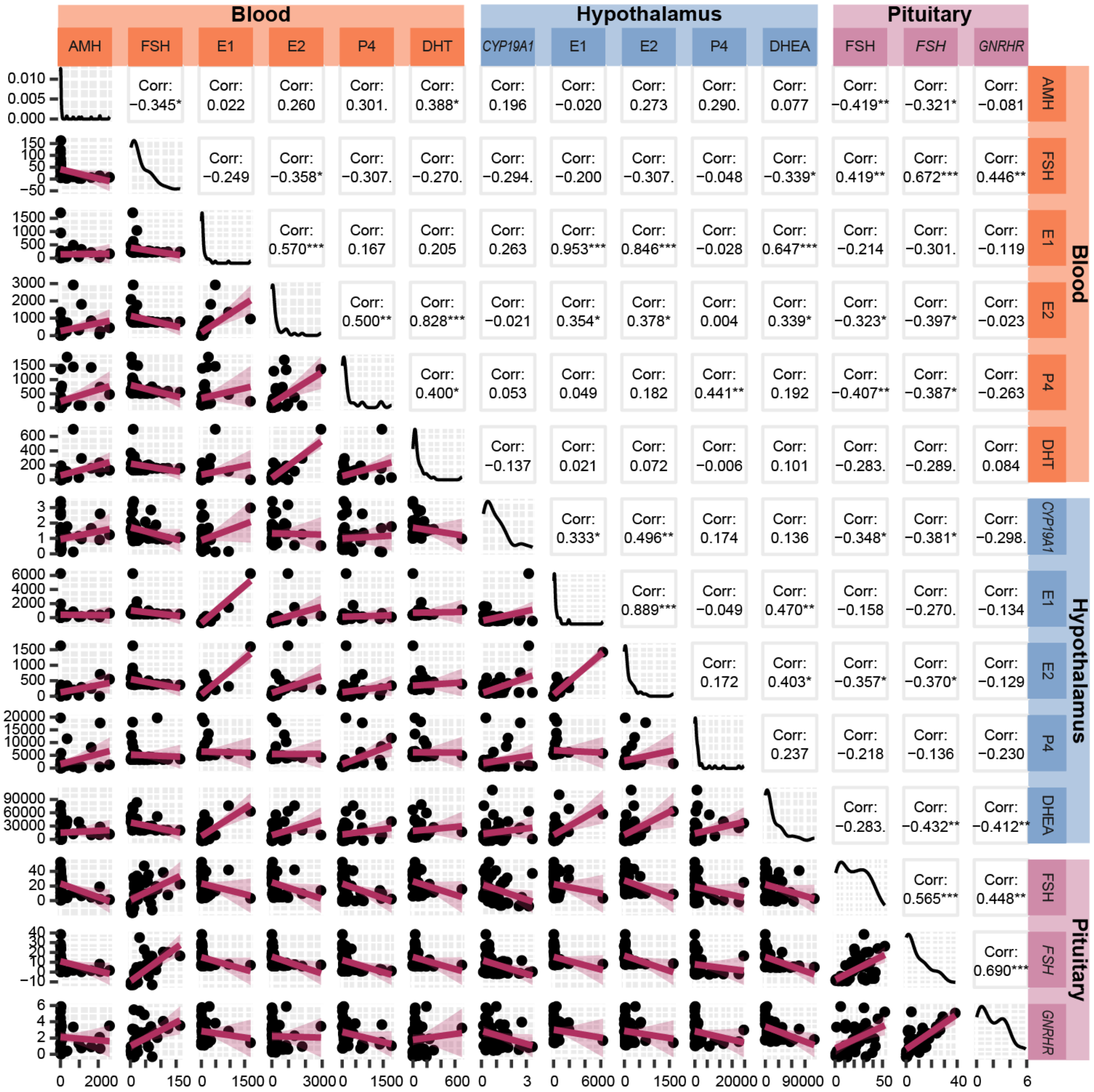

Correlation of fourteen biological measurements with significant differences between premenopausal and postmenopausal samples. The measurements were taken from blood (orange), the hypothalamus (blue), and the pituitary gland (purple). The top-left/bottom-right diagonal represents distribution of each measurement, demonstrating a non-normal distribution for all fourteen markers. Below the diagonal are scatterplots of each pair of markers with the linear regression lines imposed, showing that most markers are linearly associated. Above the diagonal are Pearson's correlation coefficients for each marker with each other. *p < 0.05, **p < 0.01, ***p < 0.001. Glossary: AMH – Anti-Müllerian hormone; DHEA – Dehydroepiandrosterone; DHT – Dihydrotestosterone; E1 – Estrone; E2 – Estradiol; FSH – Follicle-stimulating hormone (protein); *FSH* – Follicle-stimulating hormone (gene); *GNRHR* – Gonadotropin-releasing hormone receptor (gene); P4 – Progesterone.

Supplementary Figure 4

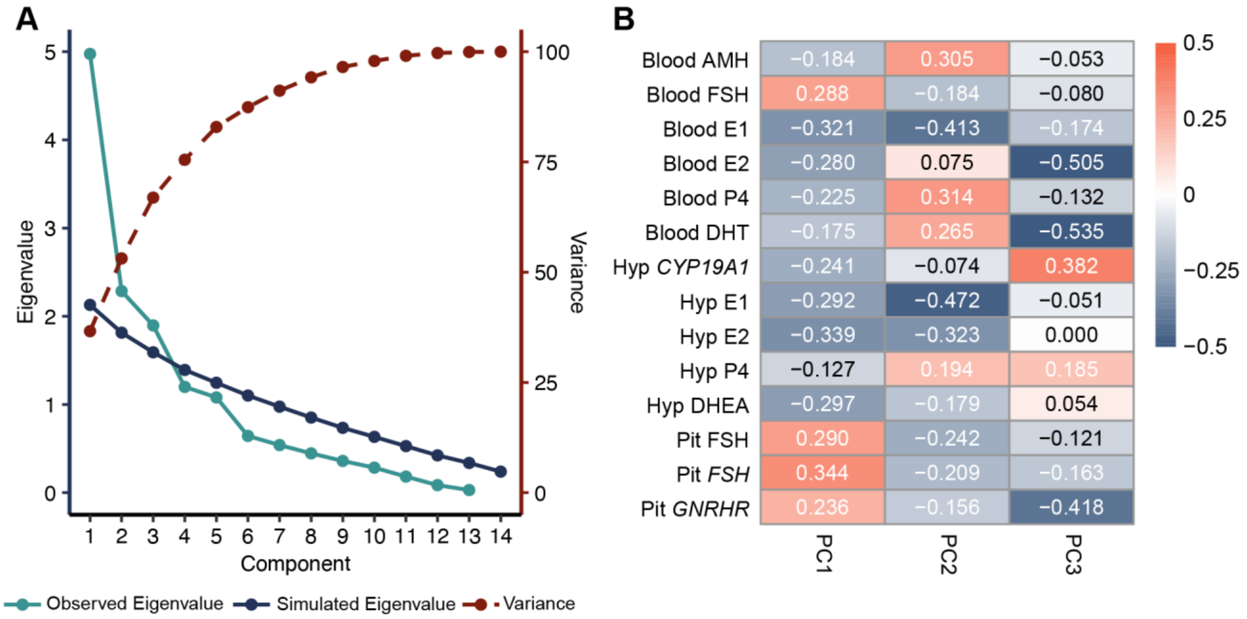

Principal component analysis for fourteen significant menopausal biomarkers. A) The fourteen significant biological markers are most appropriately combined into three components, as represented by the observed Eigenvalue (light blue line) being higher than the simulated Eigenvalue (dark blue line) with the highest variation in the dataset accounted for at three dimensions. Three dimensions account for approximately 70% of variance in the data (dashed red line). B) All fourteen significant biological measures have varying correlation with the three principal components. Glossary: AMH – Anti-Müllerian hormone; DHEA – Dehydroepiandrosterone; DHT – Dihydrotestosterone; E1 – Estrone; E2 – Estradiol; FSH – Follicle-stimulating hormone (protein); *FSH* – Follicle-stimulating hormone (gene); *GNRHR* – Gonadotropin-releasing hormone receptor (gene); P4 – Progesterone.

Supplementary Figure 5

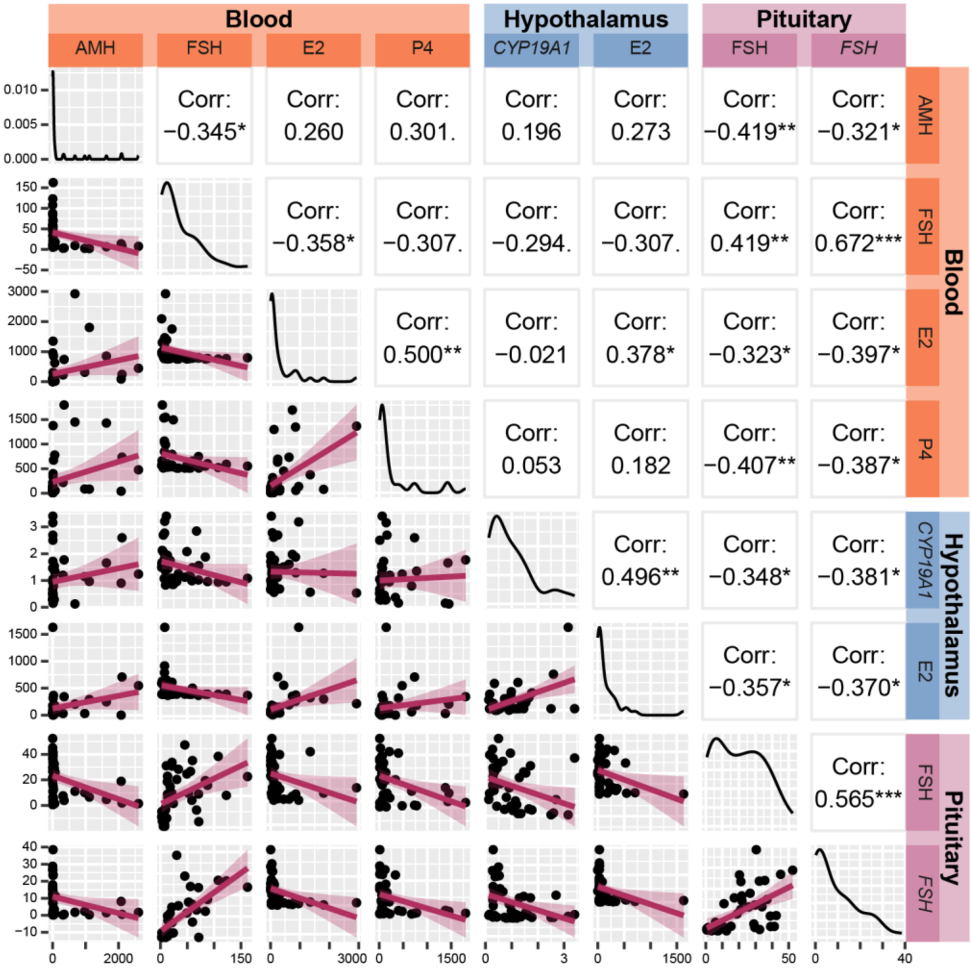

Correlation of eight biological measurements included in principal component analysis. The top-left/bottom-right diagonal represents distribution of each measurement, demonstrating a non-normal distribution for all eight markers. Below the diagonal are scatterplots of each pair of markers with the linear regression lines imposed. Above the diagonal are Pearson's correlation coefficients for each marker with each other. *p < 0.05, **p < 0.01, ***p < 0.001 Glossary: AMH – Anti- Müllerian hormone; FSH – Follicle-stimulating hormone (protein); *FSH* – Follicle-stimulating hormone (gene).

Supplementary Figure 6

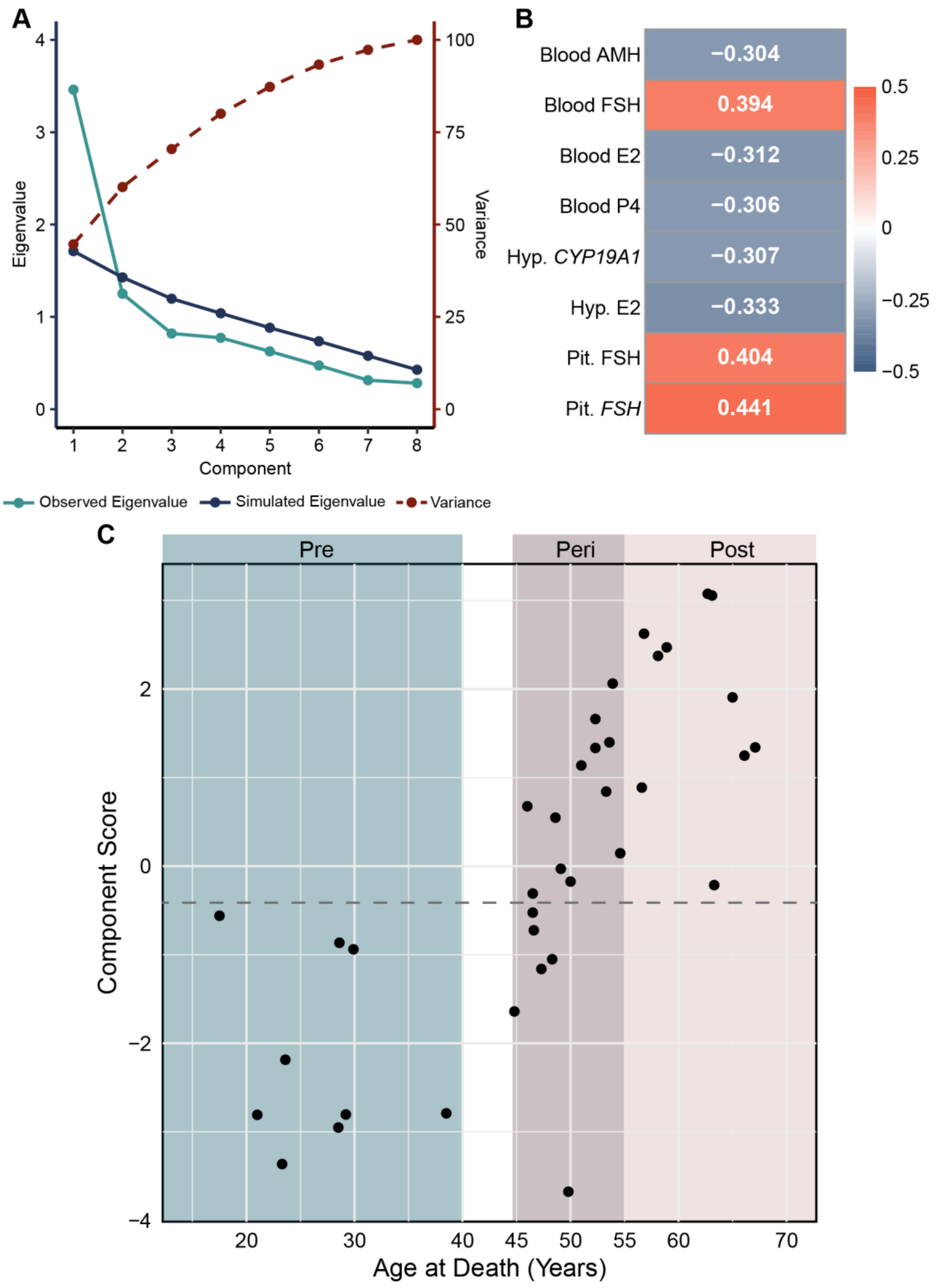

Characterization of samples based on a composite measure including hypothalamic *CYP19A1* calculated by principal component analysis. A) The eight selected biological

markers are most appropriately combined into a single component, as represented by the observed Eigenvalue (light blue line) being higher than the simulated Eigenvalue (dark blue line) only at the first component. This single component accounts for approximately 45% of variance in the data (dashed red line). B) All eight biological measures had at least moderate correlation with the final component score, as demonstrated by the absolute value of factor loadings falling between 0.3-0.5. A positive factor loading indicates that as this measure increases the component score increases, and a negative factor loading indicates that as this measure decreases the component score increases. C) Plot of chronological age vs. component score. All samples in the premenopause group had a component score of < -0.6, and all samples within the postmenopause group had a component score of > -0.2. A cutoff value to classify samples in the perimenopause group was set halfway between these two limits at -0.4 (dashed line). Glossary: AMH - AMH – Anti- Müllerian hormone; E2 – Estradiol; FSH – Follicle-stimulating hormone (protein); *FSH* – Follicle-stimulating hormone (gene); P4 – Progesterone.

Supplementary Figure 7

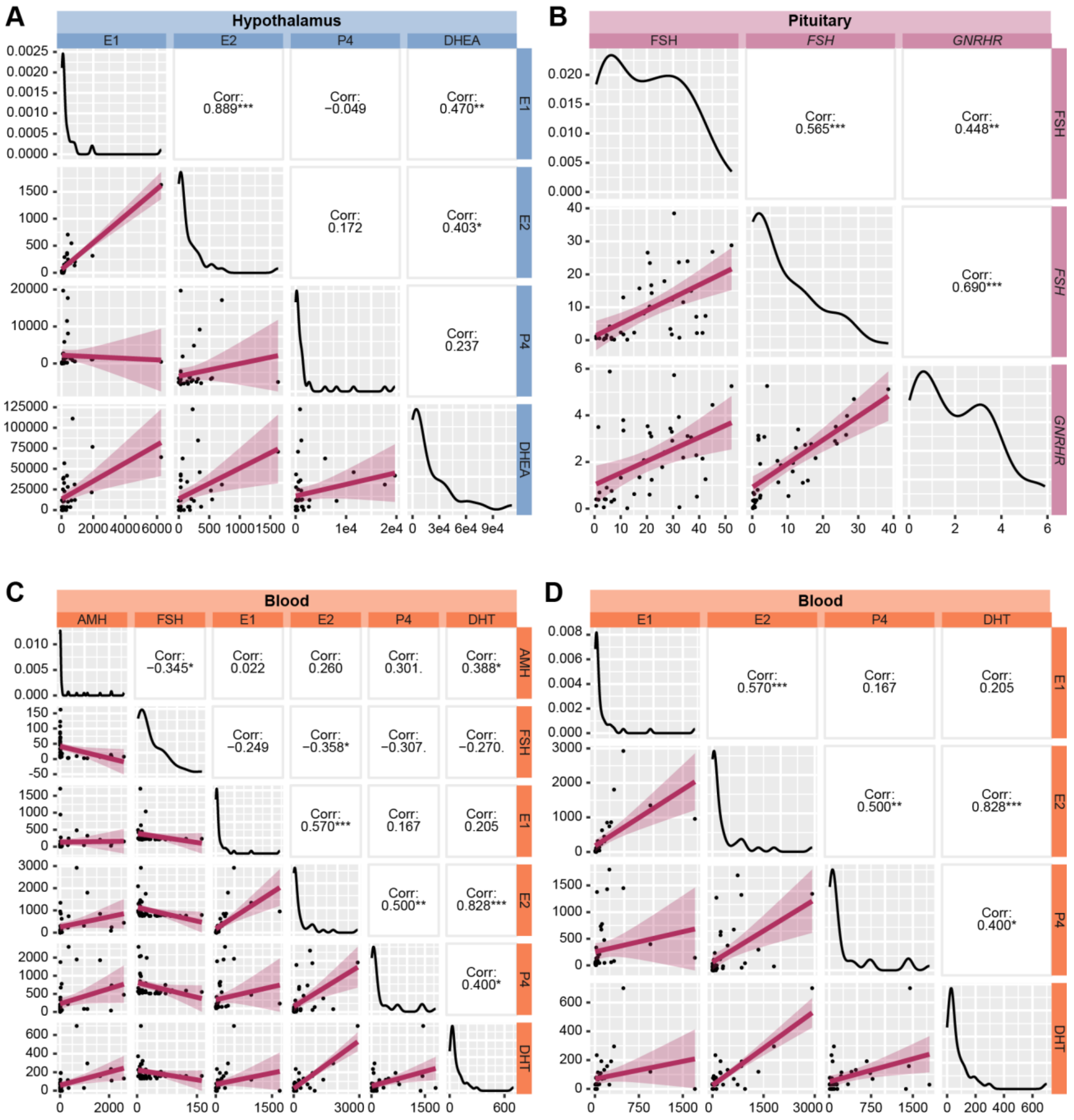

Correlations of biomarkers included in tissue-specific component score calculations. The top-left/bottom-right diagonal represents distribution of each measurement, below the diagonal are scatterplots of each pair of markers with the linear regression lines imposed, and above the diagonal are Pearson's correlation coefficients for each marker with each other for A) hypothalamus, B) pituitary gland, C) blood, and D) steroid-only blood component scores. *$p < 0.05$, **$p < 0.01$, ***$p < 0.001$. Glossary: AMH – Anti-Müllerian hormone; DHEA – Dehydroepiandrosterone; DHT – Dihydrotestosterone; E1 – Estrone; E2 – Estradiol; FSH –

Follicle-stimulating hormone (protein); *FSH* – Follicle-stimulating hormone (gene); *GNRHR* – Gonadotropin-releasing hormone receptor (gene); P4 – Progesterone.

Supplementary Figure 8

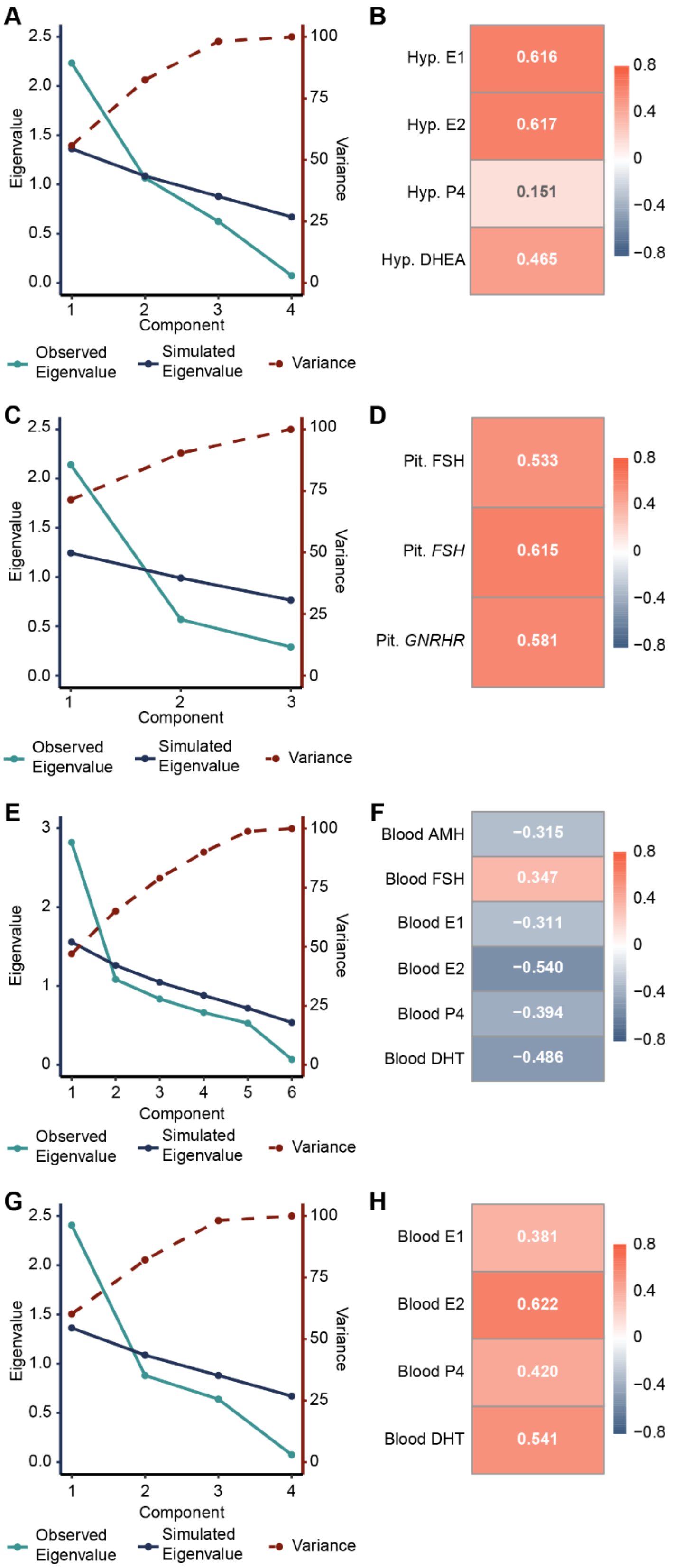

Principal component analysis for tissue-specific component scores. A) The hypothalamic markers are best combined into a single component, which accounts for approximately 56% of the variance in the data. B) All hypothalamic markers but progesterone loaded significantly into the component score. C) The pituitary markers are best combined into a single component, which accounts for approximately 71% of the variance in the dataset. D) All pituitary markers loaded significantly into the component score. E) The full blood markers are best combined into a single component, which accounts for approximately 47% of the variance in the dataset. F) All blood markers loaded significantly into the component score. G) The blood steroids are best combined into a single component, which accounts for approximately 60% of the variance in the data. H) All blood steroids loaded significantly into the component score. Glossary: AMH – Anti-Müllerian hormone; DHEA – Dehydroepiandrosterone; DHT – Dihydrotestosterone; E1 – Estrone; E2 – Estradiol; FSH – Follicle-stimulating hormone (protein); *FSH* – Follicle-stimulating hormone (gene); *GNRHR* – Gonadotropin-releasing hormone receptor (gene); P4 – Progesterone.

Supplementary Figure 9

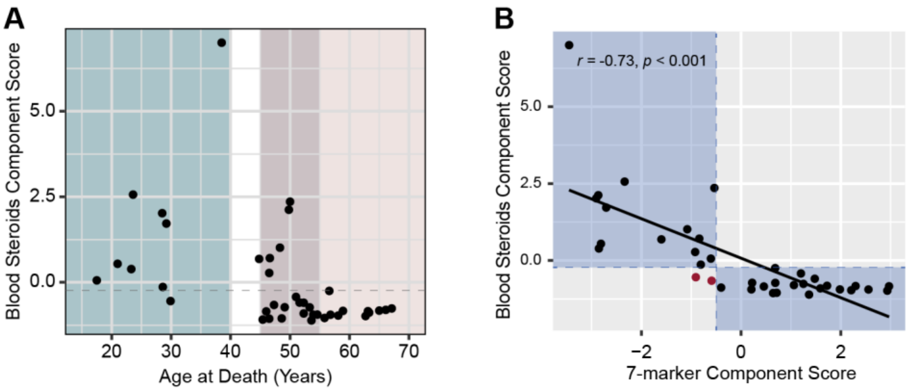

Characterization of steroids-only blood component score. A) There was some overlap in component score distribution between premenopause and postmenopause groups. With a cutoff of -0.2, all but one premenopause sample landed above the cutoff and all postmenopausal samples landed below the cutoff. B) Correlation between the blood steroids-only component score and the 7-marker component score was high, with only two samples having disagreement in classification between the two scores. Blue areas indicate where blood steroids-only tissue-specific score classification aligned with 7-marker composite measure classification, and red dots indicate samples with disagreement between the two classifications.

Supplementary Figure 10

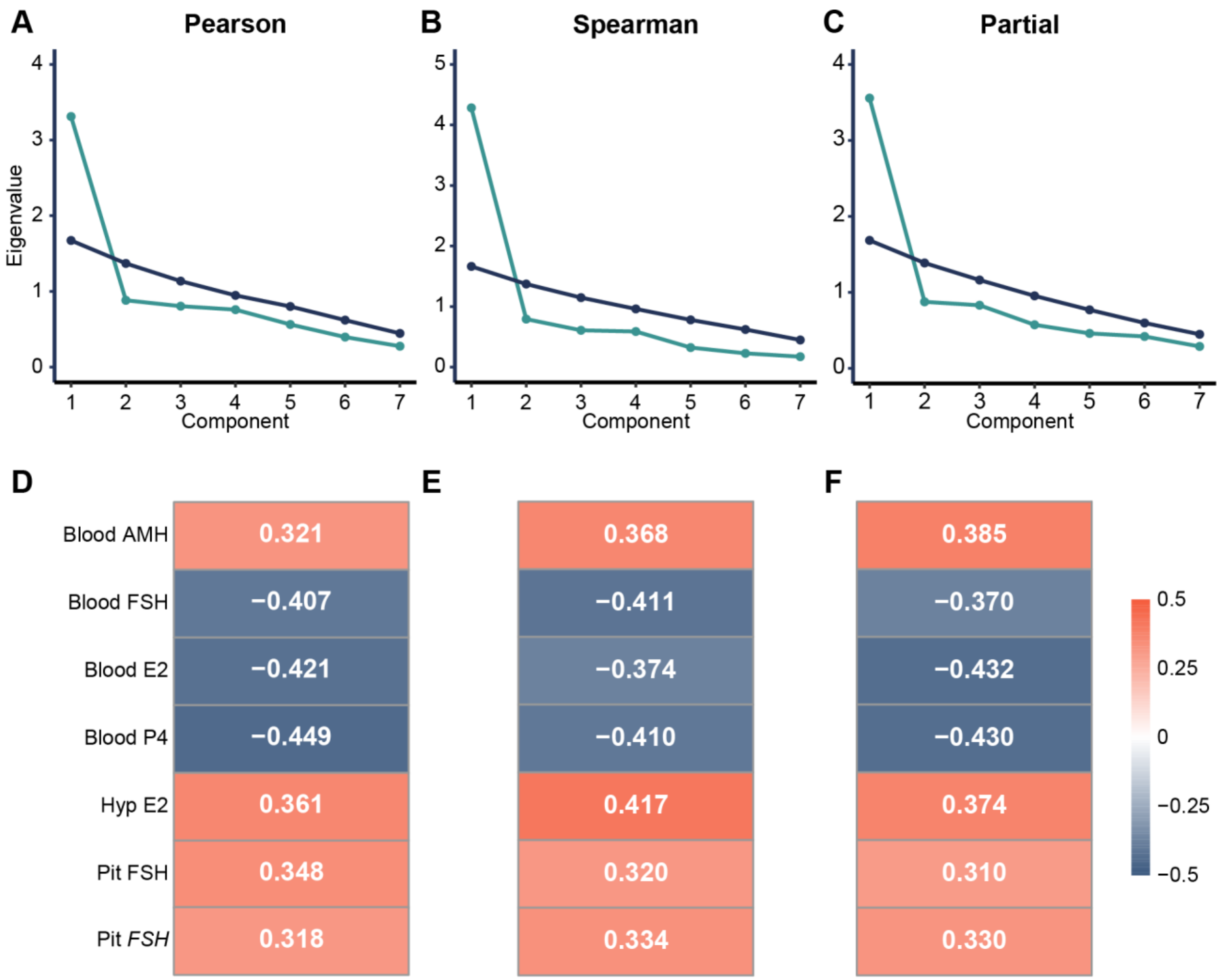

Supplemental PCA analyses with 7-marker model. A-C) Scree plots and D-F) factor loading scores for PCA models using Pearson, Spearman, and Pearson partial correlation accounting for PMI and RIN. All models have comparable factor loading scores to the final 7-marker model used for composite measure characterization of menopausal status. Glossary: AMH – Anti-Müllerian hormone; E2 – Estradiol; FSH – Follicle-stimulating hormone (protein); *FSH* – Follicle-stimulating hormone (gene); P4 – Progesterone.

Supplementary Table 2. Group values of all 40 measures analyzed by robust ANOVA. Highlighted rows indicate measures with a significant difference between groups (robust ANOVA, $p < 0.05$), with markers' significance being driven by differences between premenopause and postmenopause groups (robust post-hoc comparisons with Hochberg-Benjamini correction, $p < 0.05$). Red font highlights differences in results between the non-parametric Kruskal-Wallis and robust ANOVA analysis; note that the seven strongest markers are significant in both analyses.

	Premenopausal – Median (IQR)	Perimenopausal – Median (IQR)	Postmenopausal – Median (IQR)	F(1, 2)	p (F-test)	Pre vs. Post p (Hochberg-Benjamini)
**Glycoproteins**						
Blood						
AMH (pg/mL)	1113.34 (677.07-2078.45)	8.82 (5.52-25.83)	3.77 (0.23-9.76)	12.877	0.001	0.003
FSH (mIU/mL)	6.41 (3.02-7.63)	19.58 (11.48-56.88)	52.38 (37.91-97.81)	15.041	0.0002	0.004
Pituitary gland						
FSH (IU/mg)	5.61 (4.01-9.43)	21.85 (10.99-32.45)	30.42 (21.00-36.40)	17.995	0.00003	0.0004
**Gene expression**						
Pituitary gland						
FSH (rel. expression)	1.06 (0.56-1.75)	3.41 (1.18-12.36)	17.91 (13.63-26.70)	15.725	0.0001	0.001
ESR1 (rel. expression)	1.35 (0.70-1.77)	1.37 (1.14-2.12)	2.32 (1.13-2.74)	1.405	0.270	
GNRHR (rel. expression)	0.83 (0.48-1.90)	1.26 (0.40-3.12)	3.10 (2.24-4.19)	4.124	0.031	0.034
Hypothalamus						
CYP19A1 (rel expression)	1.17 (0.92-1.55)	0.95 (0.37-1.46)	0.33 (0.29-0.47)	5.665	0.010	0.026
ESR1 (rel. expression)	1.13 (0.81-1.28)	1.10 (0.86-1.46)	1.06 (0.60-1.51)	0.027	0.974	
ESR2 (rel. expression)	0.89 (0.74-1.20)	1.42 (1.11-1.92)	1.24 (0.50-1.45)	2.065	0.153	
KISS1 (rel. expression)	0.66 (0.39-2.21)	1.12 (0.27-2.35)	1.04 (0.64-11.72)	0.899	0.432	
GPER1 (rel. expression)	0.89 (0.73-1.48)	0.86 (0.56-1.26)	1.03 (0.68-1.59)	0.495	0.618	
PGR (rel. expression)	0.96 (0.73-1.24)	1.37 (1.03-1.63)	1.29 (0.96-1.81)	0.899	0.422	
**Steroids**						
Blood						
Aldosterone (pg/mL)	85.30 (41.02-176.97)	94.53 (19.58-284.84)	85.07 (19.75-151.09)	0.979	0.395	
Androstenedione (pg/mL)	610.13 (457.51-885.19)	429.47 (235.64-871.63)	384.45 (145.78-633.67)	1.270	0.300	

Corticosterone (pg/mL)	5144.79 (2545.65-6983.63)	3442.73 (853.14-9726.20)	5014.16 (2566.57-8408.12)	0.325	0.726	
Cortisol (pg/mL)	51731.36 (46492.11-74805.49)	62930.20 (19910.22-166069.72)	69105.31 (58189.20-93352.45)	0.985	0.391	
Cortisone (pg/mL)	6439.26 (6124.90-9978.50)	7804.68 (4845.40-17361.96)	10336.42 (7476.86-10671.95)	0.496	0.615	
11-deoxycortisol (pg/mL)	248.06 (214.18-573.49)	187.28 (124.27-849.73)	342.03 (176.99-563.14)	1.037	0.375	
DHEA (pg/mL)	4611.79 (3292.17-9312.06)	2040.58 (0.00-6776.87)	0.00 (0.00-1959.97)	5.194	0.017	0.037
DHT (pg/mL)	132.18 (109.69-234.22)	33.00 (0.00-71.75)	33.00 (30.00-33.00)	2.866	0.086	0.115
DOC (pg/mL)	28.80 (22.71-91.74)	35.28 (12.57-164.18)	48.37 (14.10-76.66)	0.175	0.841	
Estrone (pg/mL)	156.89 (49.68-245.43)	25.28 (17.33-83.75)	20.40 (17.00-33.18)	4.154	0.035	0.059
Estradiol (pg/mL)	446.39 (243.68-849.39)	65.87 (25.95-160.66)	23.98 (12.29-52.42)	4.180	0.035	0.105
17OH-progesterone (pg/mL)	279.41 (186.51-355.63)	308.56 (133.95-573.75)	127.87 (54.73-327.46)	1.673	0.210	
Progesterone (pg/mL)	477.26 (87.34-1429.87)	85.17 (44.03-242.00)	61.34 (18.40-84.41)	5.435	0.017	0.069
Testosterone (pg/mL)	478.79 (410.99-687.78)	278.80 (206.28-483.78)	327.20 (187.34-358.31)	1.883	0.180	
Hypothalamus						
Aldosterone (pg/g	144.94 (30.00-227.14)	30.00 (0.00-234.52)	166.00 (7.50-179.71)	0.041	0.960	
Androstenedione (pg/g)	999.76 (736.27-1430.89)	406.47 (188.67-1263.65)	357.15 (91.37-523.30)	0.443	0.648	
Corticosterone (pg/g)	8867.67 (3472.11-18187.80)	3216.42 (600.00-20691.34)	5226.10 (2686.39-9429.71)	0.325	0.726	
Cortisol (pg/g)	20433.88 (15166.19-37791.22)	15865.83 (8906.07-101410.12)	21081.38 (14417.18-69108.67)	0.776	0.473	
Cortisone (pg/g)	1834.45 (847.12-2941.47)	1335.27 (600.00-4220.00)	1330.79 (600.00-1697.93)	3.068	0.071	
11-deoxycortisol (pg/g)	485.86 (148.83-926.77)	534.81 (60.00-2987.06)	375.34 (137.10-734.68)	1.572	0.236	
DHEA (pg/g)	25405.86 (11806.28-37697.34)	3600.00 (3600.00-16709.99)	0.00 (0.00-9000.00)	2.543	0.104	0.132
DHT (pg/g)	180.00 (180.00-180.00)	180.00 (180.00-180.00)	180.00 (180.00-180.00)	0.001	0.999	
DOC (pg/g)	146.48 (44.57-334.23)	47.28 (18.00-472.30)	108.48 (18.00-209.10)	0.910	0.421	
Estrone (pg/g)	371.62 (169.70-570.48)	126.86 (89.58-324.00)	30.00 (30.00-102.57)	3.248	0.056	0.104
Estradiol (pg/g)	236.72 (73.57-328.72)	30.00 (30.00-178.19)	30.00 (30.00-30.00)	6.097	0.010	0.037
17OH-progesterone (pg/g)	1138.62 (648.20-2170.98)	390.91 (180.00-2970.40)	457.92 (180.00-544.06)	0.338	0.717	
Progesterone (pg/g)	882.31 (497.73-6396.58)	434.69 (147.53-1278.97)	139.68 (60.00-307.58)	1.314	0.303	0.604
Testosterone (pg/g)	422.80 (273.02-873.55)	281.15 (203.68-564.39)	234.33 (152.93-365.10)	0.084	0.920	

Supplementary Table 5: Primer sets used for qRT-PCR in the pituitary and hypothalamus.

Gene	RefSeq Accession	Forward	Reverse
GAPDH	NM_001357943	ACA ACT TTG GTA TCG TGG AA	AGG GAT GAT GTT CTG GAG AG
TBP	NM_001172085	CAA ACC CAG AAT TGT TCT CC	TAC ATG AGA GCC ATT ACG TC
PSMC4	NM_153001	TCA CTA GCA AGA TGA ACC TC	ATT CCA CTC TCC TGA CAG AT
CYC1	NM_001916	TCT TAG AGT TTG ACG ATG GC	ATC ATC AAC ATC TTG AGC CC
UBE2D2	NM_181838	TTG CTC GGA TCT ACA AAA CA	TTT CTC TTT CAG AGT TCC CC
EIF4A2	NM_001967	AGT GGT TTT TCG GAT CAT GT	AAT CTC ATT CCA GTT GCT CT
CYP19A1	NM_001347256.2	ATC CTT TTG GCA TGT TCC	CCT TAG AAG TTT GAG CCC
ESR1	NM_001122742	CTT TCT CCT GCC CAT TCT AT	TGT GCT TAC TCC TTC CTA GT
ESR2	NM_001291712	CTA GAA CAC ACC TTA CCT GT	AGA AGT GAG CAT CCC TCT TT
KISS1	NM_002256	GCC AGG TGG TCT CGT CA	TGA GAA GAG GCA GGT CCT AGA
GPER1	NM_001505	GTG CCA GGA CAA TGA AAT AC	TTT ACA TCA TCA CCG CAG TT
PGR	NM_001202474	GCC GTG CTC AAG GAG G	GAT GCT TCA TCC CCA CAG
FSH	NM_001382289	GAA GAA TGT CGT TTC TGC AT	TGG GTC CTT ATA CAC CAG AT
GNRHR	NM_000406	AAT CAT CTT CAC CCT GAC AC	AGT CCA GCA GAC AGT AAA TG
